# From raw to scaled human spinal cord morphometry: An original system for identifying complex anatomical patterns

**DOI:** 10.1371/journal.pone.0359730

**Published:** 2026-10-26

**Authors:** G. Glaudot, Z. Delbaere, C. Ferenc, C. Behets, E. Pircalabelu, A. Jankovski

**Affiliations:** 1 Pole of Morphology, Institut de Recherche Expérimentale et Clinique (IREC), Université Catholique de Louvain (UCLouvain), Brussels, Belgium; 2 Institute of Neuroscience (IoNS), UCLouvain, Brussels, Belgium; 3 Institute of Statistics, Biostatistics and Actuarial Sciences (ISBA), UCLouvain, Louvain-La-Neuve, Belgium; 4 Department of Neurosurgery, Clinique Sud Luxembourg Vivalia, Arlon, Belgium; King Faisal University, SAUDI ARABIA

## Abstract

Understanding the segmental anatomy of the human spinal cord remains challenging due to its interindividual variability and the lack of standardized definition of a spinal cord segment. In this human cadaveric study, we dissected 40 spinal cords, identified and measured the length, thickness and width of each segment comprised between C2 to S5. This extensive dataset enabled us to characterize the distribution and variability of the measurements, as well as to conduct ratio and correlation analyses. By converting segment length measurements into an original coordinate system, and thickness and width values into scaled data relative to easily identified anatomical features, we showed that L4 and C6 can be considered relevant landmarks of the cadaveric SC. We also divided the sample into three subgroups according to the total SC length: a short group (n = 9), an intermediate group (n = 15), and a long group (n = 16). This distinction revealed unexpected morphological features regarding the transverse dimensions, showing that variations in total SC length did not result in proportional changes in average segment thickness or width. Overall, these new morphometric findings highlight the gross morphological complexity of a generally oversimplified structure and provide additional anatomical landmarks, improving our understanding of SC cadaveric anatomy.

## Introduction

Understanding the segmental anatomy of the spinal cord (SC) remains challenging due to its high inter-individual morphometric variations and methodological subtleties regarding segment limits across studies [1,2]. Furthermore, SC imaging is an arduous task due to its small cross-sectional dimensions, its surrounding anatomical environment, and its physiological motion [3]. These considerations hinder both standardization of data and the interpretation of measurements. The variable topographical relationship between SC segments and their corresponding vertebrae, as well as the increasing discrepancy between them in caudal direction, also contributes to the difficulty of drawing firm conclusions [2]. Despite their clinical importance, few studies have sought to establish landmarks and references that enable to identify patterns that would allow to overcome the complexity of the SC.

Recent imaging studies suggested new approaches to break away from the imprecise and unreliable localization of SC segments based on vertebral levels. These approaches include the use of new neuroanatomical landmarks [4,5] or the automatic segmentation of the nerve rootlets, enabling to define the segment limits [6]. However, these promising techniques address the cervical and most rostral thoracic segments but do not cover the entire SC length. In addition, they require high-resolution scans and expert knowledge to guide the analysis despite the increasing automation of the measurements and of the segmentation processes, which cannot readily be applied in daily clinical practice.

Several cadaveric studies have focused on segmental [1,7–10] or regional [11] measurements, contributing to a better understanding of the different subdivisions of the SC. However, these measurements did not systematically include the entire SC length, and the reference points used to define the segment limits differed substantially [2]. In a first postmortem study, we identified several topographical markers based on segment length, thickness, and width [10]. These landmarks consisted in both extremities of the cervical and lumbar enlargements, mostly based on width and thickness variations, respectively. The thoracic region was characterized by stable thickness and width values associated with high variations in segment length. We also concluded that the region between L2 and S5 exhibited minimal length variation, regardless of the sex of the subjects. Subsequently, we conducted a second study focusing exclusively on the regional lengths of the SC and vertebral column [11]. These data confirmed our initial hypothesis that the thoracic region was the most important contributor to total SC length variation and that the length of the lumbosacral region was remarkably stable across subjects. Therefore, we suggested using the total SC length as a new approach to explain regional length variations, rather than using other criteria, such as sex or the position of the conus medullaris in the vertebral canal.

The present cadaveric study aims to expand our dataset and further explore the SC anatomy by determining new localization markers which, through statistical analyses, will supplement these preliminary results. To achieve this, we measured the length, thickness, and width of segments C2 to S5 of 40 SCs and conducted a preliminary analysis based on these raw data, including characterization of the distribution and variation of the measurements, as well as different ratios and correlation analyses. Subsequently, we converted the raw length data into a novel segment coordinate system, as well as thickness and width data into scaled measurements, based on easily identified anatomical references. Based on our second study [11], the sample was then divided into three groups according to total SC length. All analyses made on raw data were conducted on scaled data and the outcomes from both methods were compared.

This dual approach allowed us to identify reliable anatomical landmarks on cadaveric specimens, thus complementing the observations made just on raw measurements from our previous studies, and unveil some aspects of the human SC anatomical complexity. Further investigations must now be conducted to confirm whether these morphometric findings can be confirmed with in vivo MRI measurements. Ultimately, this approach is designed to be translated into an MRI context, and to provide practitioners with improved localization tools for their daily SC MRI.

## Materials and methods

### Bodies

This study was conducted on 40 European cadavers (21 females) embalmed with a zinc chloride solution (mean age at death: 84.8 ± 7.9 years), including 32 subjects from a previous study which already explored segmental measurements [10]. Twenty subjects of this first dataset were already used in a subsequent exploration which studied the lengths of spinal and vertebral regions [11]. To reach the 40 cadavers of the present study, 8 new subjects were included alongside the 32 subjects from the first dataset. All cadavers had been voluntarily donated to the Laboratory of Anatomy of UCLouvain (Brussels, Belgium) through the accredited biobank BB 190051, with written informed consent provided prior to death for educational and scientific use. In accordance with Belgian federal legislation governing the use of human body material for research purposes (Act of 19 December 2008, as amended by the Act of 23 February 2022), the use of cadavers supplied through an accredited biobank does not require additional project-specific approval by an institutional ethics committee. All procedures were conducted in accordance with relevant Belgian legislation and internationally accepted ethical principles, including the Declaration of Helsinki, as applicable.

### Experimental protocol

The extraction of the 8 supplementary specimens followed the same procedure as in our previous studies [10,11]. Samples were excluded in case of SC or vertebral column surgery, significant vertebral deformity, neurodegenerative disease and any neurological conditions capable of altering the general morphology of the SC. The entire vertebral column and posterior cranial fossa were extracted from each body. Each vertebra was identified, as well as each spinal nerve exiting the intervertebral foramen starting at the first cervical vertebra and verified at the twelfth thoracic vertebra to avoid labeling mistakes related to transitional vertebrae. The spinal nerves T1, L1, and S1 were labeled on both sides. The pedicles of C2–L5 vertebrae were sectioned. In addition, the foramen magnum, C1 vertebra, and sacrum were sectioned on their posterior aspect. The vertebral arches were removed, exposing the posterior aspect of the spinal dura. SCs and their dura were extracted by sectioning the meningo-vertebral ligaments at the posterior aspect of the vertebral bodies. Each SC was straightened on a polystyrene plate, then the dura was opened on its anterior aspect and over its entire length, to expose the anterior rootlets. Since left and right rootlets were not symmetrically distributed, we determined the point located at equal distance from the emergence of the lowermost and uppermost rootlets of 2 successive spinal nerves on each side. A virtual line was drawn between the left and right points, and its intersection with the SC anterior midline was defined as the limit between 2 adjacent segments, as illustrated in supporting figure (S1 Fig). This procedure was repeated for each segment of the 40 SCs. Each intersegmental limit was marked with an entomological needle at the anterior SC surface, and the 29 segments (C2 to S5) were delineated. The C1 and coccygeal segments were not considered due to the difficulty of identifying their limits. Although subjective, this method seemed to us the most anatomically appropriate. Indeed, it considers the asymmetry between the left and right rootlets and assumes that consecutive segments are contiguous elements, without spaces between them as in some imaging studies [12,13]. Additionally, this study measured the transverse dimensions (thickness and width) in the middle of each segment, rather than at its rostral extremity, as conducted in other methods [1,2].

### Measurements

#### Raw data.

The length, thickness and width of the 29 segments of the 40 SCs were measured independently by two examiners with a digital caliper. The anterior length of each segment was defined as the distance between its rostral and caudal limits. The total SC anterior length was calculated by adding all individual segment lengths. Thickness and width were measured in the middle of each segment and were defined as the antero-posterior and latero-lateral diameters, respectively (Fig 1A). Ratios between total SC length and thickness as well as width of each segment, were calculated in order to better understand the relationship between longitudinal and cross-sectional measurements.

**Fig 1:**
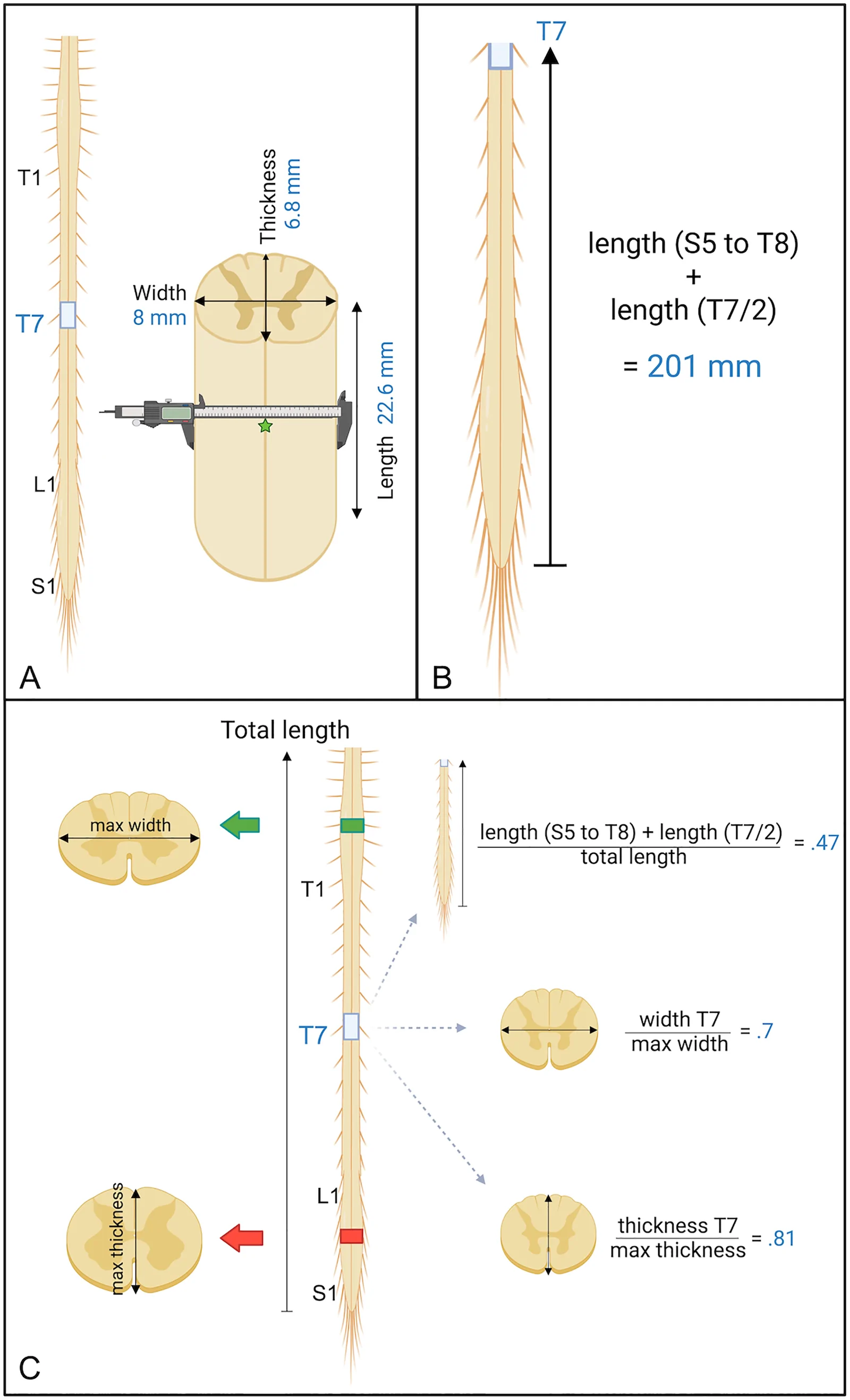
Measurement methods. A. Raw data consisting of length, thickness and width measurements (in mm) of the 29 segments (C2 to S5). The mean values for the T7 segment are provided (in blue) as an example. Transverse dimensions were measured in the middle of each segment (green star). B. Conversion of the individual segment lengths into a caudo-rostral length coordinate system. C. Original approach consisting of converting the raw length coordinates into scaled length coordinates (0 to 1), as well as a thickness and width ratio, expressed in reference to the thickest segment of the lumbosacral enlargement (=1), and the widest segment of the cervical enlargement (=1), respectively.

A first transformation was applied to convert the individual segment lengths into a caudo-rostral length coordinate system. This step presented the data cumulatively, starting from the caudalmost rootlet of S5 (representing level 0) and ending at the rostralmost rootlet of C2 (representing the total SC length). The coordinate of a given segment was obtained by adding the length of the caudal segments and half of the length of the given segment (Fig 1B).

#### Scaled data.

To better compare the morphological features of the SCs among the 40 subjects and due to the high variability of the data, we considered an innovative approach designed to smooth variations through scaling of the caudo-rostral length coordinate, as well as relative width and thickness, with each value expressed as a ratio. Consequently, in this scaling method, measurements are no longer expressed in millimeters but as values comprised between 0 and 1. With this scaling of the raw data, we target extensions of our analyses to future studies that are non-invasive, MRI-based and for which implementing our suggested approach is straightforward. As a direct consequence, it also bypasses the need for knowing the absolute segment measurements. We acknowledge that the cadaveric approach is information-rich but acquiring the same amount of information would be more difficult in non-invasive techniques. We detail below how the scaling for length, thickness and width is performed throughout our analysis:

**Caudo-rostral length coordinate**. Scaling the data consisted in a second transformation, that converted the length coordinate system expressed in millimeters, into a scaled model expressed as a ratio, from 0 (corresponding to the caudalmost rootlet of S5) to 1 (corresponding to the rostralmost rootlet of C2). The coordinate of a given segment was obtained by adding the length of the caudal segments and half of the length of the given segment and reporting this sum to the total length (Fig 1C).

**Scaled thickness and width**. Segment thickness and width were also expressed as a ratio, with 1 corresponding to the maximal thickness and width measured in the lumbosacral enlargement and in the cervical enlargement, respectively (Fig 1B). This methodological choice was guided by our previous observations, which showed that the lumbosacral enlargement was characterized primarily by an increase in thickness, whereas the cervical enlargement was dominated by an increase in width.

Following a preliminary analysis of the entire sample (n = 40), further investigations were conducted by dividing the sample into three subgroups based on total SC length, as was done in our previous study [11]. This subdivision consisted of a group of short (n = 9), intermediate (n = 15), and long SCs (n = 16), as further detailed and illustrated in Fig 5 in the Results section.

### Statistics

We used an intraclass correlation coefficient (ICC) test to assess the consistency of inter-examiner measurements for the three dimensions, separately. As the ICC were systematically greater than 0.8, we concluded that there was a strong correlation between the two examiners and used the mean values as final raw data on which statistical analyses were performed.

The coefficient of variation (CV), defined as the ratio between the empirical standard deviation and the empirical mean, expressed data dispersion relative to the mean values.

The Kruskal-Wallis H test compared the medians between 3 groups of SCs sorted according to their total length, to highlight significant differences between groups. In a second step, the post-hoc Dunn’s test helped determine the groups that were significantly different.

Spearman’s ρ coefficient and its associated nullity hypothesis test were used for all correlations.

The significance level for determining the rejection of a null hypothesis was set to the value α = 10% prior to any analysis. We chose a significance level of 10% as it is an exploratory study with, from a statistical, testing power perspective, a small number of subjects, although this is already substantial for a cadaveric study. Rejection of the null hypothesis was concluded if the calculated p-value associated with the statistical test was below the threshold α.

When multiple comparisons were performed, multiplicity corrections to the p-values were applied using the Holm–Bonferroni method. Although this approach is conservative, we chose this family-wise error rate control to highlight only strong, relevant statistical effects and reduce potentially false positive conclusions.

All statistical analyses and figures were produced using R (v 4.4.1). Figs 1 and 10 were created using BioRender (https://BioRender.com).

## Results

First, this section will consider the entire sample by presenting the results obtained with the raw and scaled datasets for each dimension. Subsequently, we will introduce length-based grouping, present group differences, and replicate the correlation analysis by group.

### Entire sample

#### Length.

**Raw data**. The empirical distribution of the 29 segments was first plotted with boxplots (Fig 2-A1). This method corroborated the results of our previous studies [10,11], as well as the results found in the literature [1,2], showing higher median values and variations in the thoracic region than in the other regions. The CV estimated with the raw data showed fluctuations around 15 for segments comprised between C2 and L5 (Fig 2-A2). It then increased strikingly in the region S1 to S5, reflecting high variations and low mean values in the sacral segments.

**Fig 2:**
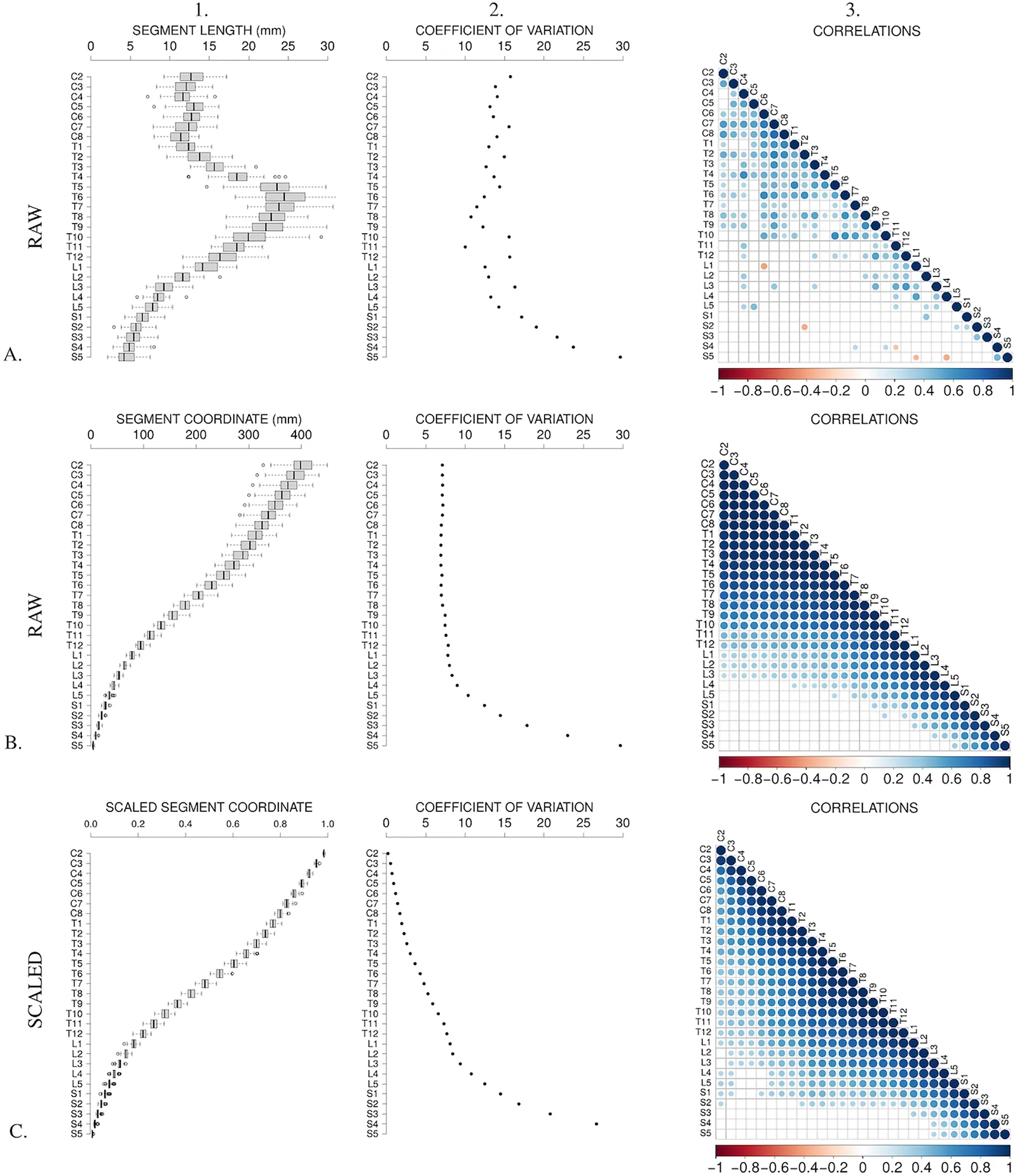
Segment length analysis. 1. Boxplots. 2. Coefficient of variation. 3. Correlation heatmap. A. Raw data. B. Segment coordinate system. C. Scaled data.

The rank-based correlation analysis showed weak associations between nearby segments in the cervicothoracic region, which then dissipated in the caudal direction (Fig 2-A3).

The same analysis on the caudo-rostral coordinate system resulted in incremental median values from S5 to C2 (Fig 2-B1). It also resulted in an increase of the standard deviation (SD) towards the most rostral segments, as well as a smoothing of the CV curve with stable values around 7 from C2 to L3 (Fig 2-B2). The CV then increased in the caudal direction in the region from S1 to S5. As expected, the correlation analysis revealed decreasing correlations between segments as we reach the caudal extremity of the SC, due to the cumulative effect of this alternative method. The region between L4 and S5 appeared uncorrelated from most of the rostral segments.

**Scaled data**. While the boxplots presented a similar curve to the raw length coordinate system, the scaling reduced the discrepancy in variation between the lower and upper SC levels (Fig 2-C1). The SD was maximal in the mid-thoracic region and gradually decreased in the rostral and the caudal direction. The CV curve presented an incremental evolution, starting nearly at 0 in C2 and presenting a more pronounced slope in the lumbosacral region (Fig 2-C2). The correlation analysis exhibited a comparable pattern to the raw segment coordinates, with a more extensive yet less strongly correlated region of segments (Fig 2-C3). The strength of the correlations between segments did not gradually decrease in caudal direction but presented a more balanced pattern between consecutive segments.

#### Thickness.

**Raw data**. The curve shape of the median values in the boxplots was also consistent with prior descriptions, including our previous findings [1,2,7,10]. Decreasing values were found from C2 until the beginning of the thoracic region, which was characterized by stable values. Then, an increase was observed from L1 toward the lumbosacral enlargement, followed by a sharp decrease until S5 (Fig 3-A1). The CV pattern showed slight variations comprised between 10 and 15 from C3 to L3, followed by a decrease in L4. It then increased strikingly up to S5, again showing high variations and low mean values for sacral segments (Fig 3-A2). Moderate correlations were observed between nearby segments and dissipated as the physical distance increased. The sacral segments exhibited a moderately strong correlation among them but were uncorrelated with the other spinal segments (Fig 3-A3).

**Fig 3:**
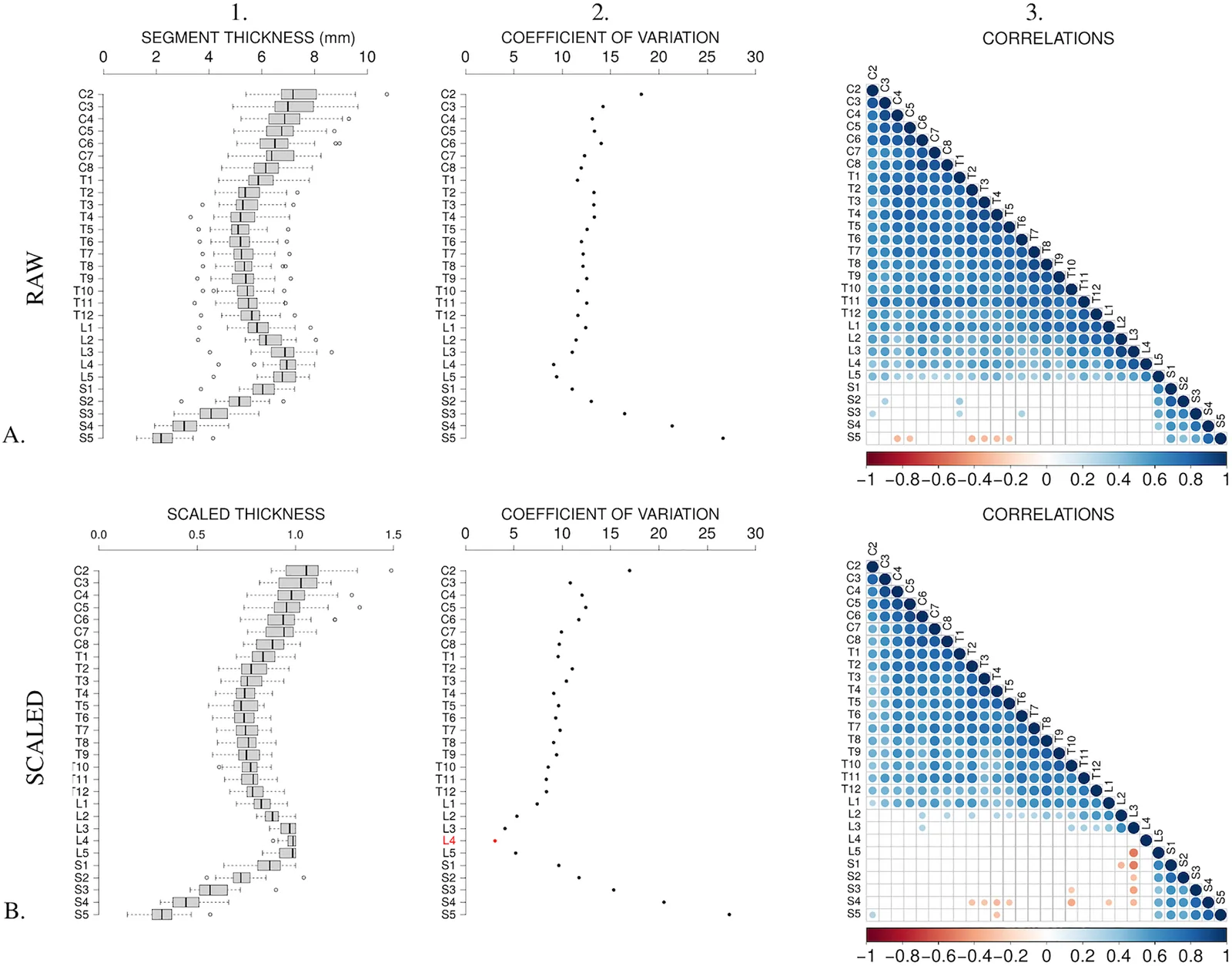
Segment thickness analysis. 1. Boxplots. 2. Coefficient of variation. 3. Correlation heatmap. A. Raw data. B. Scaled data.

**Scaled data**. Scaling did not affect the general shape of the boxplot curve but presented less outliers than the raw method (Fig 3-B1). Interestingly, the L4 segment presented very few variations as revealed by the aspect of its boxplot. The CV curve presented comparable stability to that of the raw data but accentuated the lumbar enlargement and highlighted L4 as the segment with the less variations (Fig 3-B2). The scaling method slightly attenuated the strength of the correlations while enlarging the extent of the uncorrelated region up to the first lumbar segments (Fig 3-B3). It also revealed an absence of correlation for L4 and punctual negative correlations. The rest of the correlation heatmap did not show notable differences from the raw model.

#### Width.

**Raw data**. As for the segment length and thickness, the median values of the width showed a similar pattern as previously described in the literature [1,2,7,10]. The highest values were found in the cervical enlargement while stable values were found in the thoracic region. Moreover, a slight increase was found in the lumbosacral enlargement, before a sharp decrease up to S5 (Fig 4-A1). The CV curve exhibited stable values around 10 between C2 and L4 segments before increasing in the region from L5 to S5 (Fig 4-A2). The correlation analysis showed moderate associations between segments from C2 to L4. The strength of the correlation then decreased for the sacral segments (Fig 4-A3).

**Fig 4:**
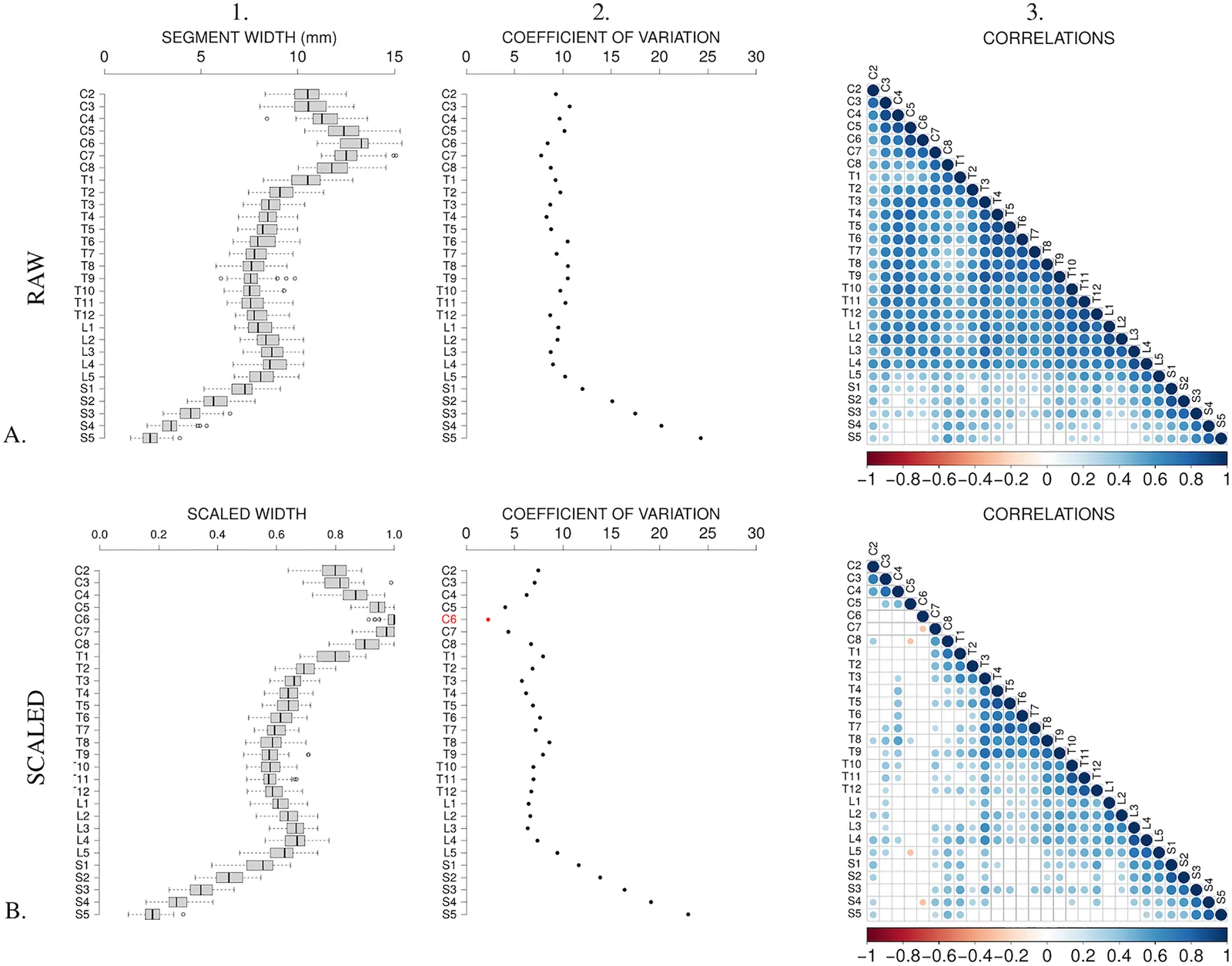
Segment width analysis. 1. Boxplots. 2. Coefficient of variation. 3. Correlation heatmap. A. Raw data. B. Scaled data.

**Scaled data**. Similarly to the analysis of thickness, the scaling method did not modify the boxplot curve but presented fewer outliers than the raw method (Fig 4-B1). The C6 segment presented very few variations as revealed by the aspect of its boxplot and the low value of the CV (Fig 4-B1 & 4-B2). As with thickness, scaling slightly reduced the strength and extent of the correlations. In addition, an absence of correlation in C5 and C6, as well as the appearance of a few negative correlations were also observed after scaling (Fig 4-B3). More notably, the correlation pattern appeared sparser with the scaled data than when the raw data were used.

### Length-based grouping

In a previous study [11], we determined that the total SC length was a relevant criterion to sort our sample in order to explain regional length variations. We decided here to use the same method and divided the 40 specimens into 3 groups according to the total SC length, as follows (Fig 5):

- a first, *Short*, group including the 9 shortest specimens, ranging from 327 mm to 379 mm (mean 357.7  ± 17 mm),
- a second, *Intermediate*, group with 15 specimens of intermediate length, from 385 mm to 400 mm (mean 393.2  ± 5.4 mm),
- a third, *Long*, group consisting of the 16 longest specimens, from 405 mm to 449 mm (mean 423.8  ± 13.4 mm).

**Fig 5:**
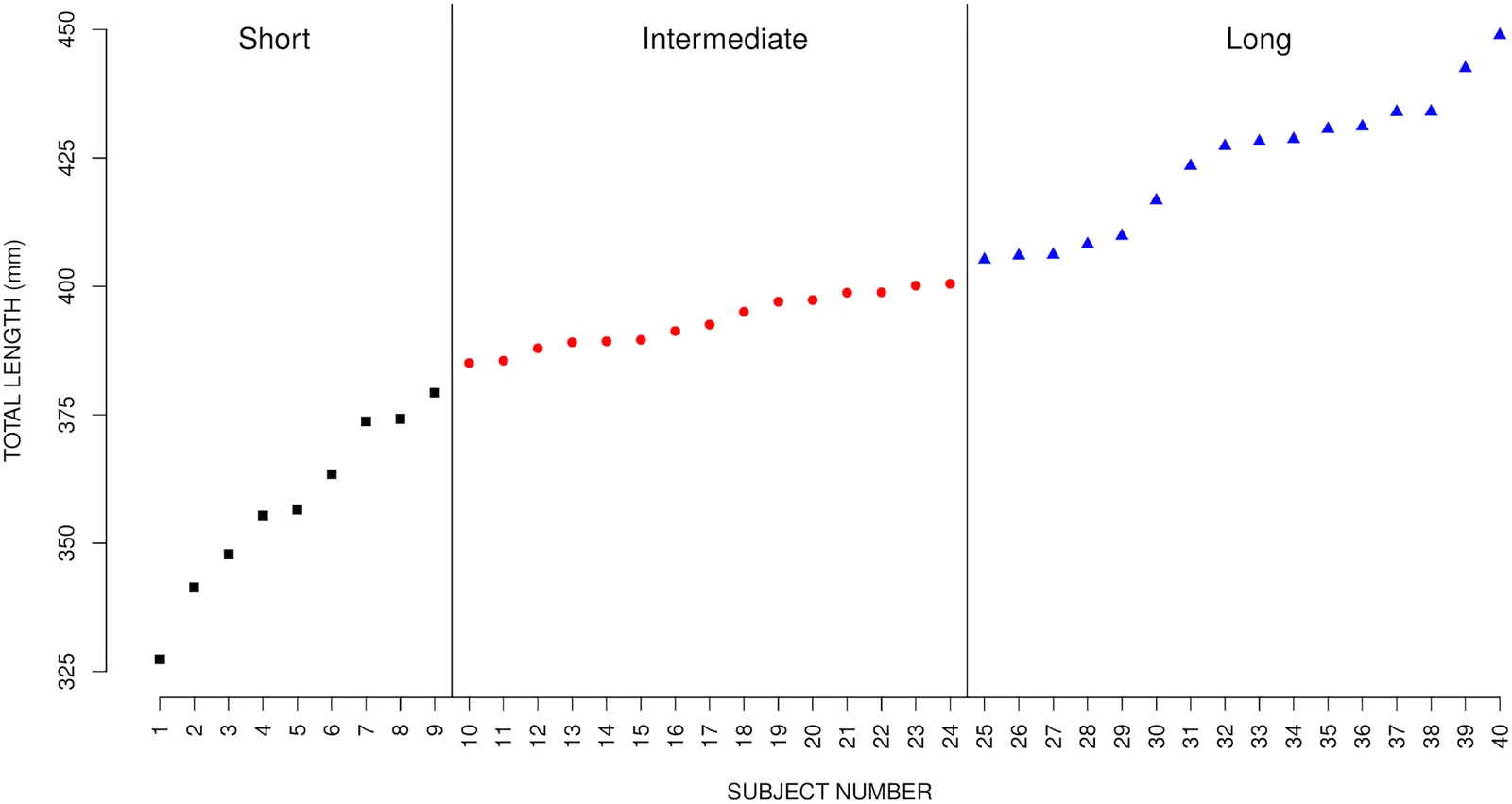
Grouping of the sample based on total SC length. Left: Short group (n = 9, mean = 357.7 mm ± 17 mm); Middle: Intermediate group presenting limited length variation (n = 15, mean = 393.2 mm ± 5.4 mm); Right: Long group (n = 16, 423.8 mm ± 13.4 mm).

Among the 3 groups, the *Intermediate* one served as the reference group as it showed limited length variation, with the lowest standard deviation.

### Median comparison between groups

We compared the median length, thickness and width of each segment between the 3 groups and identified subsequently the groups between which significant differences were found for each dimension (Fig 6). All the analyses first compared the *Intermediate* group to the *Long* group and then to the *Short* group. In the following sections, we describe and focus on the general trends that seemed relevant for our study.

**Fig 6:**
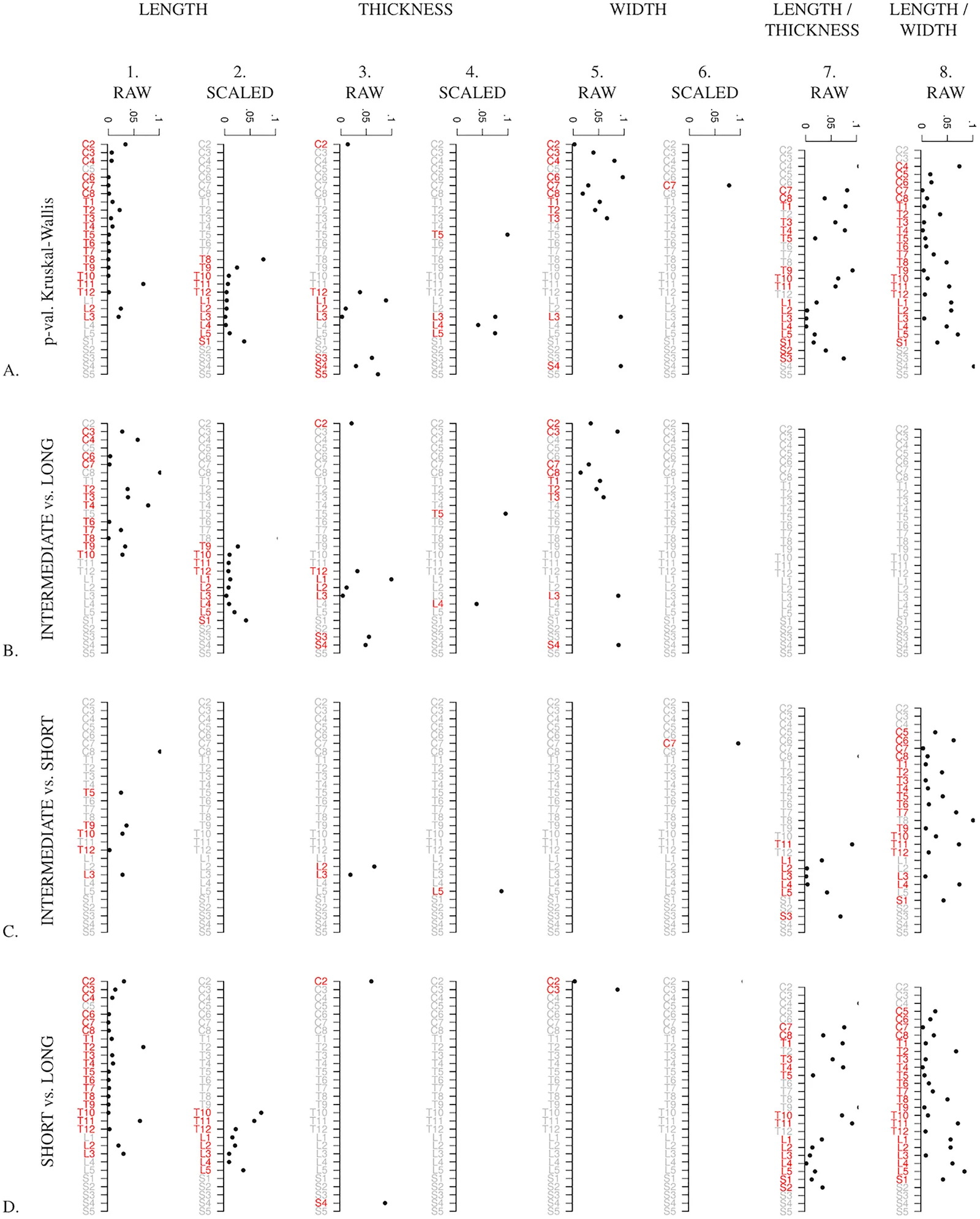
Group differences for the segment length, thickness and width. A. Kruskal-Wallis H test. B-C-D. Post hoc Dunn’s tests successively presenting the raw and scaled data for the segment length (1, 2), thickness (3, 4) and width (5, 6). Equality across the 3 groups for ratios between the total SC length and the segment thickness and width were also tested (7 and 8, respectively). Significant differences in red.

#### Length.

The null hypothesis of equal median length between the 3 groups was rejected for almost all segments comprised between C2 and L3 (Fig 6-A1). Unsurprisingly, the majority of these rejections were driven by differences in median length between the *Short* and *Long* groups (Fig 6-D1) and to some extent, between the *Intermediate* and *Long* groups (Fig 7-B1).

**Fig 7:**
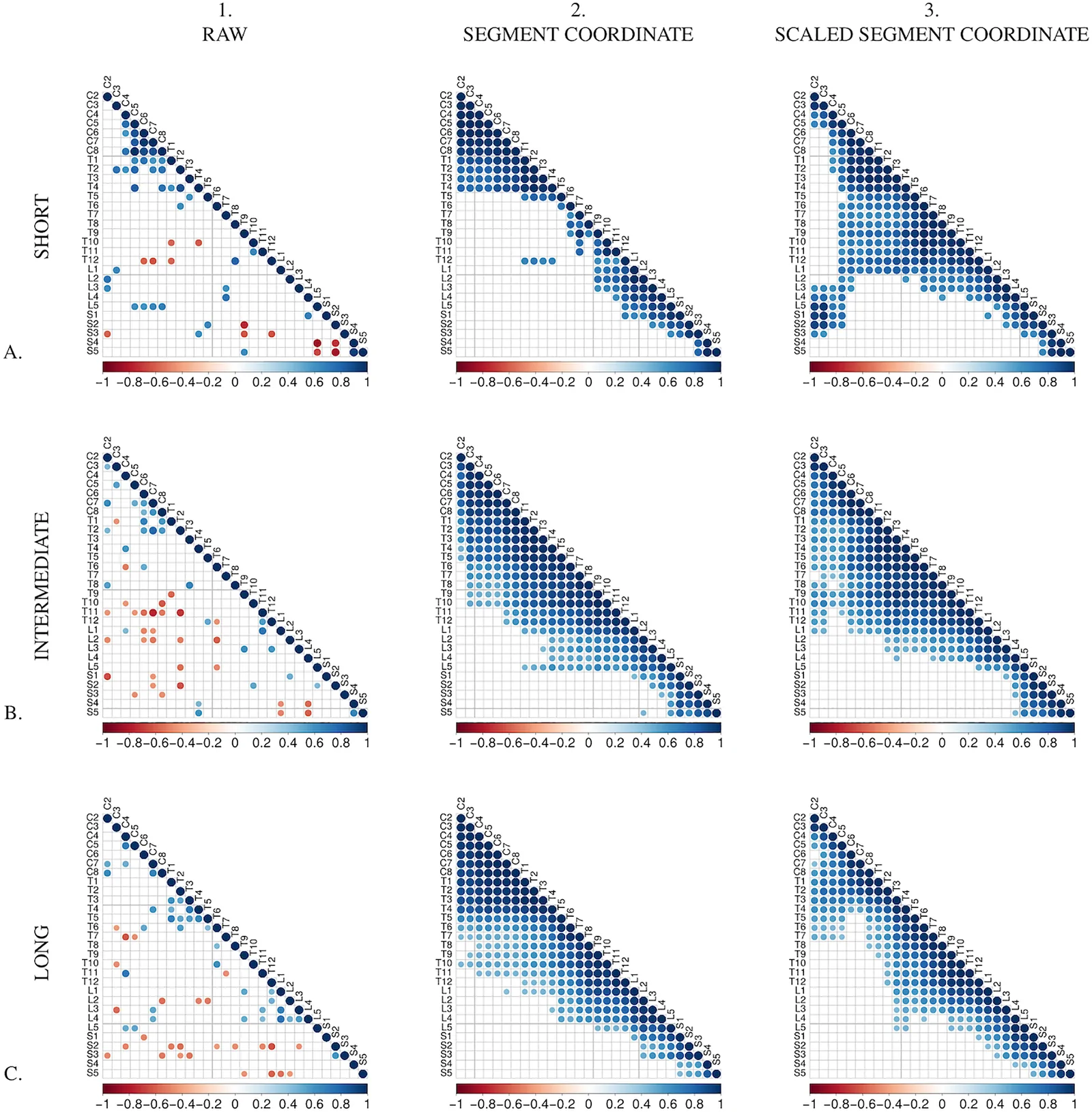
Correlation analysis of the segment length. 1. Raw data. 2. Segment coordinates. 3. Scaled segment coordinates. A. Short group. B. Intermediate group. C. Long group.

After scaling, significant differences between groups were found in all segments comprised between T8 and S1 (Fig 6-A2). As in the raw data, the large majority of these differences were driven by differences between *Intermediate* and *Long* groups (Fig 6-B2) and between *Short* and *Long* groups (Fig 6-D2). Quite surprisingly, for the scaled data, differences were mostly observed in the lumbar and preceding thoracic segments, while for the raw data, the differences were concentrated much more in the cervical and nearby thoracic regions.

#### Thickness.

The null hypothesis of equal median thickness between the 3 groups was rejected in C2, T12 to L3, and S3 to S5 (Fig 6-A3). Almost all rejections were driven by differences in median thickness between the *Intermediate* and *Long* groups (Fig 6-B3) but punctual significant differences in thickness were also observed between the *Intermediate* and *Short* groups (Fig 6-C3) and between the *Short* and *Long* groups (Fig 6-D3).

Although significant differences in raw thickness were mostly found in the first lumbar and last sacral segments, the scaling concentrated the differences in the L3-L4-L5 segments (Fig 6-A4). Only very few, punctual significant differences were found when comparing the groups (Fig 6-B4, C4, D4).

#### Width.

The null hypothesis of equal median width between the 3 groups was mainly rejected in most segments in the region C2 to T3 (Fig 6-A5). Similarly to the thickness analysis, almost all rejections were driven by differences in width between the *Intermediate* and *Long* groups (Fig 6-B5), but significant differences were also observed in the C2 and C3 segments between the *Short* and *Long* groups (Fig 6-D5).

The scaling appeared to eliminate all significant differences in width, except in C7, between the *Intermediate* and *Short* groups (Fig 6-C6).

#### Ratios.

Since we expected the transverse dimensions (thickness and width) to behave differently depending on the total SC length, and to better understand the relationship between these variables, we calculated the ratios and tested whether there were statistical differences between the 3 groups (Figs 6-A7, A8). This analysis was only performed using raw data as for the scaled data, the total SC length is, by construction, always = 1.

**Total SC length / segment thickness**. Overall significant differences between groups were found in segments comprised between C7 and S3, except for a few thoracic segments (Fig 6-A7). Yet again, most rejections were driven by differences between the *Short* and *Long* groups (Fig 6-D7) and to a lesser extent, between the *Intermediate* and *Short* groups (Fig 6-C7).

**Total SC length / segment width**. Overall significant differences between groups were found in all segments from C4 to S1 (Fig 6-A8). Consistently across segments, also for this ratio, the *Short* group displayed a different anatomical pattern relative to the *Long* and *Intermediate* groups.

**Segment width / segment thickness.** No significant difference was observed between any of the groups, which potentially suggests that the ratio of these transverse dimensions fluctuates much less across different SC lengths.

### Correlation comparison between groups

We deepened the analysis by inspecting pairwise correlations between the length, width and thickness values of all segments in the 3 groups.

#### Length.

Correlation analysis of the raw values revealed inconsistent, sparse correlations and failed to provide information in any of the 3 groups (Figs 7-A1, B1, C1). In contrast, when considering the raw length coordinate system, the *Intermediate* and *Long* groups revealed similar heatmaps with strong correlations between consecutive segments which then decreased with the distance caudally from lower thoracic segments (Figs 7-B2, C2). The *Short* group seemed to conserve strong correlations only between segments between C2 and T4, as well as moderate correlations between consecutive segments comprised between T9 and S1 (Fig 7-A2).

Few differences were found between correlations in the raw segment coordinates and the scaled values for the *Intermediate* and *Long* groups (Figs 7-B2, B3, C2, C3). Surprisingly, the scaling had a direct consequence highlighting more profoundly the different anatomical features of the short SCs relative to the long and intermediate ones, as the correlation pattern of the *Short* group before and after scaling was more different from that of the other 2 groups.

#### Thickness.

Correlation analysis of the raw data revealed a similar pattern among the 3 groups, with strong correlations between segments from C2 to L2 (Figs 8-A1, B1, C1). A nearly total absence of correlation was observed caudal to these segments, except between the sacral segments, where moderate correlations persisted. The *Short* group exhibited stronger correlations than the other two groups; punctual negative correlations were found in the *Short* and *Long* groups.

**Fig 8:**
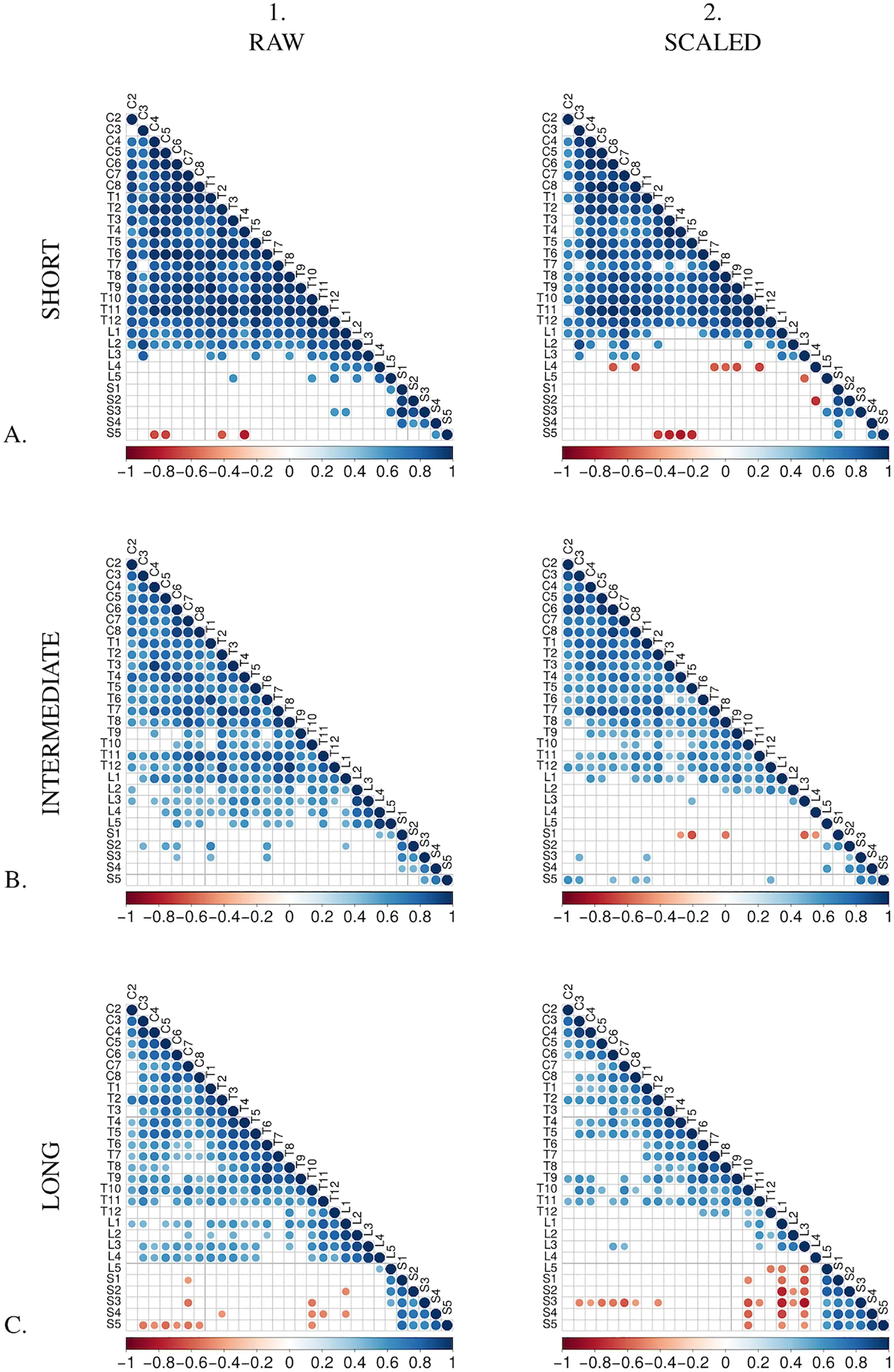
Correlation analysis of the segment thickness. 1. Raw data. 2. Scaled data. A. Short group. B. Intermediate group. C. Long group.

Scaling data did not change the general pattern of the heatmap except in the *Long* group for which slightly more negative correlations appeared (Fig 8-C2) for segments comprised between T10 and L3, and the sacral segments (Figs 8-C1, C2).

#### Width.

Quite differently from the thickness analysis, when focusing on width measurements, correlation analysis of the raw data revealed a stronger contrast among the 3 groups, with the *Intermediate* and *Long* groups showing a much larger number of positive correlations than the *Short* group (Figs 9-A1, B1, C1).

**Fig 9:**
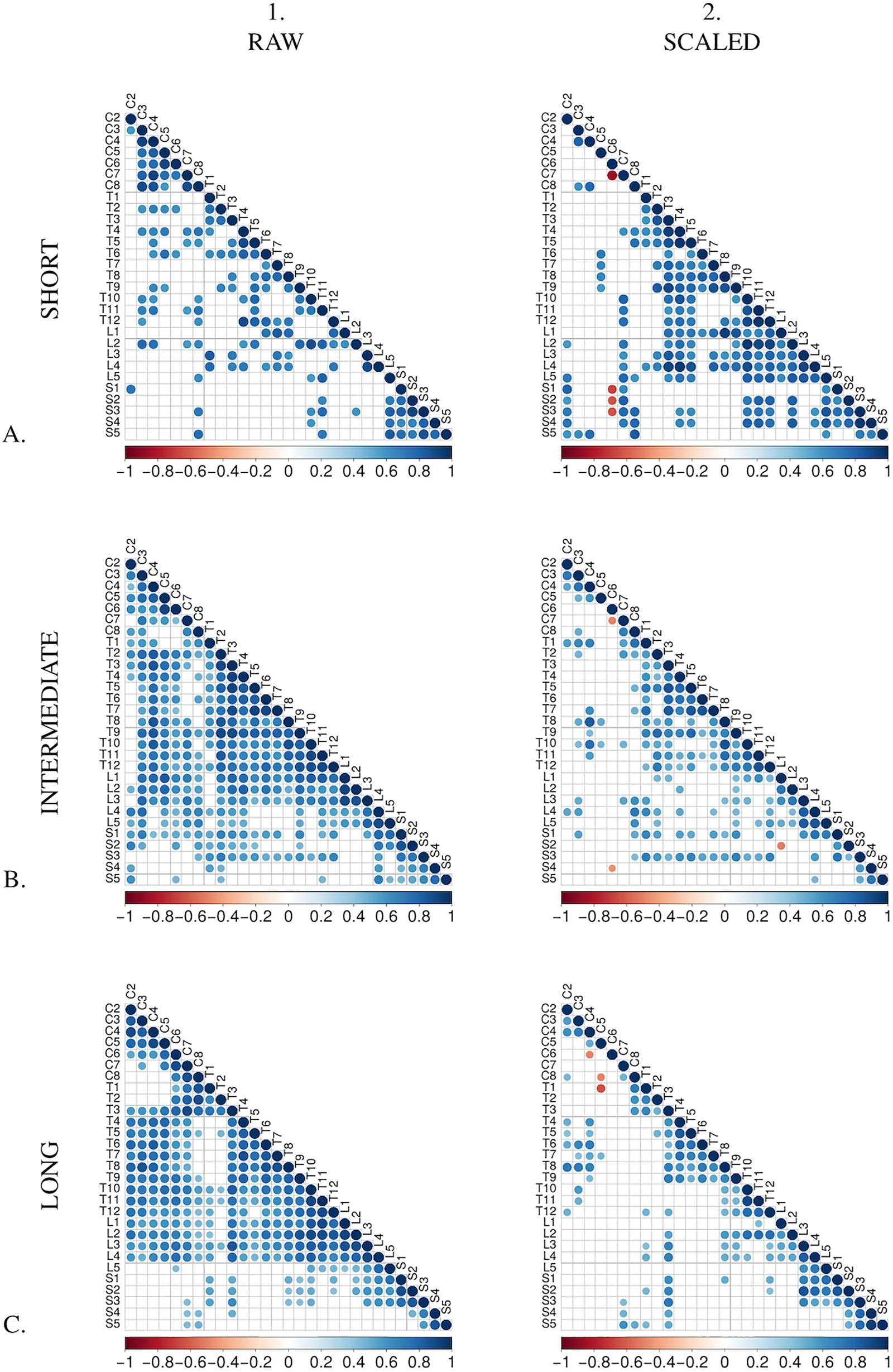
Correlation analysis of the segment width. 1. Raw data.2. Scaled data. A. Short group. B. Intermediate group. C. Long group.

The scaling eliminated multiple correlations in the *Intermediate* and the *Long* groups (Figs 9-B2, C2) but identified more correlations in the thoracolumbar region in the *Short* group (Fig 9-A2). In addition to a loss of correlation between C5-C6 and the other segments, negative correlations emerged after the scaling, particularly in C6 for the *Short* and *Intermediate* groups and in C5 for the *Long* group.

## Discussion

The high variability in SC anatomy within the population induces intractable difficulties in interpreting localization with daily clinical practice imaging techniques [14]. Data standardization is also prevented by heterogeneous methodologies, ranging from the types of exploration (MRI, X-ray myelography, CT scans, postmortem examination) to the lack of widely recognized definition of the segment limits, or the exact position of the measurements [2].

This cadaveric study aimed to supplement our previous morphometric descriptions [10,11] which included 32 and 20 specimens, respectively. The significance level was deliberately set at α = 10% as the present study was designed as an exploratory analysis aimed at identifying morphometric patterns and potential anatomical landmarks rather than testing a limited number of prespecified hypotheses. Given the limited sample size, the subdivision of the sample into three groups, and the large number of statistical comparisons, this threshold was considered to provide an appropriate balance between sensitivity to potentially relevant patterns and control of false-positive findings. Family-wise error rate was controlled through correction for multiple comparisons. Accordingly, findings for which the family-wise error rate was at most 10% were interpreted as exploratory trends requiring further validation, particularly in vivo, before the proposed landmarks can be considered robust for imaging applications.

The analysis of raw measurements of the three dimensions of each segment in the present study was logically consistent with values found in our previous reports. We also explored the adequateness of our length-based grouping and designed an original method using scaled rather than raw data to overcome high data variability. We thus attributed three scaled measurements to each segment: a length coordinate relative to a caudal reference point, a scaled thickness with respect to the thickest segment of the lumbosacral enlargement (mainly dominated by an increase in thickness [10]), and a scaled width with respect to the widest segment of the cervical enlargement (dominated by an increase in width [10]). The conversion of the raw measurements into scaled data was thus only conducted on the extended dataset of 40 specimens and consisted of a distinctive and innovative approach compared to our preliminary findings.

Our raw measurements of individual segment lengths were consistent with previous studies [1,2,10], which allowed us to validate our methodology. Conversion of these raw length data into the novel rostro-caudal coordinates markedly improved data coherence and structure (Fig 2B). The simple visual analysis of the boxplots and the curve shape clearly demonstrated a representation of the data that appears more coherent with visual anatomical observations than using the raw measurements, highlighting the greater variability of the thoracic segment length than the lumbosacral one. It also reduced the CV fluctuations around the baseline, without affecting its increase in the sacral segments, due to low mean values. The stability of the CV curve in the more rostral segments can be explained by high variations associated with high mean values, due to the construction of the method. However, the correlation heatmap mostly reflected the coordinate system effect of the measurements and did not provide relevant information.

Scaling the segment coordinate data provided another form of regularity, with coherent and anatomically consistent data as compared to the raw data methods (Fig 2). The scaled boxplots provided a representative overview of the morphometric features and reduced the number of outliers in the sample, suggesting a more homogeneous and better-balanced distribution. Scaling resulted in a broader pattern of correlations between consecutive segments. It reduced the cumulative effect, presenting a version of the data that appeared more relevant than with both raw approaches. It should be noted that none of these approaches affected the CV increase of the sacral region. Indeed, this behavior is due to the high variability associated with lower mean values of these segments, across the 3 dimensions. These results may appear contradictory to our previous study [11], which showed that the raw value of the sacral region length was particularly stable despite high variations in the total SC length. However, the variations found in this analysis are relative to the small mean length of each sacral segment. Therefore, these two observations are not contradictory, but rather refine our understanding of the conus medullaris anatomy.

Scaling the segment thickness and width based on the thickest and widest segments of the lumbosacral and cervical enlargements irrespective of which they were, respectively, allowed us to account for anatomical variability and identified 2 salient segments: L4, revealed by thickness and C6 revealed by width. Interestingly, these segments did not systematically correspond to the maximum values found in the literature (expressed as raw measurements in millimeters). Kameyama et al. reported the exact same curve profiles as ours in a study of 12 Japanese subjects, highlighting L4 and C6 as maximal values [9]. Meanwhile, Ko et al. found the maximal thickness and width at L3 and C5, respectively, in 15 Korean adults, suggesting that their peak values were displaced one level upward compared to ours. In a recent review, Frostell et al. reported very similar thickness and width values between L3 and L4 and between C5 and C6, respectively, with minimal advantages for L3 and C5 [2]. These differences among the studies likely reflect different methodologies regarding the references used to define segment limits, as well as the exact position within the segment at which the transverse dimensions were measured [2]. In our opinion, the larger sample size, the homogeneity of our dataset, and the scaling method used in this study, along with the analysis of the CV, boxplots, and correlation heatmaps, provided a more robust and sophisticated approach to identify the segments with the highest values. Indeed, L4 distinctly appeared as the segment with the lowest CV value of thickness, the narrowest boxplot with the highest median, as well as the most uncorrelated segment (Fig 3B). Although the median values of the L3 and L5 boxplots were very close to those of the L4, the CV analysis highlighted this segment due to its low variation. Similarly, after scaling, C6 appeared as the segment with the lowest CV value, the narrowest boxplot with the highest median, as well as the most uncorrelated segment (Fig 4B). The thickest segment of the lumbosacral enlargement can thus reasonably be considered to be L4, and the widest segment of the cervical enlargement can reasonably be considered to be C6, although our data show this is not systematically true. These observations were revealed through anatomical features, not imposed criteria, and can therefore be regarded as reasonably reliable morphometric landmarks of the SC anatomy.

The second strategy used to deepen the analysis was to divide the 40 subjects into 3 groups. This process was also implemented in our previous study [11] which revealed that the total SC length, rather than the sex of the subjects or vertebral column features, was the best criterion for predicting SC regional length variations. As a result, we showed that the thoracic region of the SC was the primary contributor to total SC length variation, while the cervical region contributed to a smaller extent. In contrast, the lumbosacral region exhibited a constant length across all subjects. Thus, we deemed it appropriate to sort the subjects of the present study using the total SC length criterion, since length was still a studied parameter, albeit at the segmental and not regional level. In this context, we considered the *Intermediate* group to be the reference group since it exhibited the least amount of variability, as previously observed [11].

The Kruskal-Wallis test on scaled length coordinates of the 3 groups revealed significant differences more clearly, with a cluster in a smaller region between T8 and S1 (Fig 6-A2). Post hoc Dunn’s tests specified that these differences were observed when comparing the *Long* group with the other two groups. This suggests that the proportionality of T8-S1 region as compared to the total SC length is not respected. It also indicates that the variations in segment coordinates from C2 to T7 are proportional to the total SC length and that this proportionality is lost from T8 to S1, demonstrating a differential contribution of the segments in the variations of the total SC length. It should be noted that, in accordance with our previous study [11], this was not the case for the raw data, which rather showed length fluctuations from C2 to the mid-lumbar segments (Fig 6-A1). Significant differences observed in the raw data analysis reflect proportionality between segments, while significant differences obtained with the scaled data analysis indicate a loss of proportionality. The same analysis performed on scaled thickness and width data strikingly reduced the number of significant differences between the 3 groups. This suggests that scaling homogenized the data by reducing variability (Figs 6−2, 4, 6).

The ratio of the total SC length to the segment thickness (Figs 6-7C, 7D) revealed significant differences between the *Short* and the other groups. On the opposite, there was no difference between the *Intermediate* and *Long* groups, which suggests that these dimensions might behave proportionally between these 2 groups. The same results were obtained for the ratio of the total SC length to the width of each segment. This led us to compare the means of the total SC length, and the mean thickness and width of all the segments between the 3 groups with 3 schematic cylinders presenting elliptical sections (Fig 10). Their dimensions—the height, the minor and the major axes of the ellipses—represent the average total SC length, the average thickness and the average width of the 29 segments together, respectively. Switching from the *Intermediate* to the *Long* group requires an increase of all 3 dimensions of the volume. This increase can be understood intuitively if we consider that longer SCs are found in taller subjects, who have more extended territories that need to be innervated. However, switching from the *Intermediate* group to the *Short* group does not decrease the transverse dimensions that, on the contrary, slightly increase. Intuitively, it could be hypothesized that the transition from an intermediate SC to a short one should reduce every dimension of the SC. However, counterintuitively, the segment thickness and width do not decrease proportionally and even slightly increase, suggesting a compensatory effect of the length reduction. It is as if there were a physical limit beyond which width and thickness could not shrink any further, potentially due to the need to preserve function. This interesting observation deserves further investigation in an *in vivo* context to determine whether this phenomenon is also observed, or whether it may be partly due to structural deformation, as discussed below.

**Fig 10:**
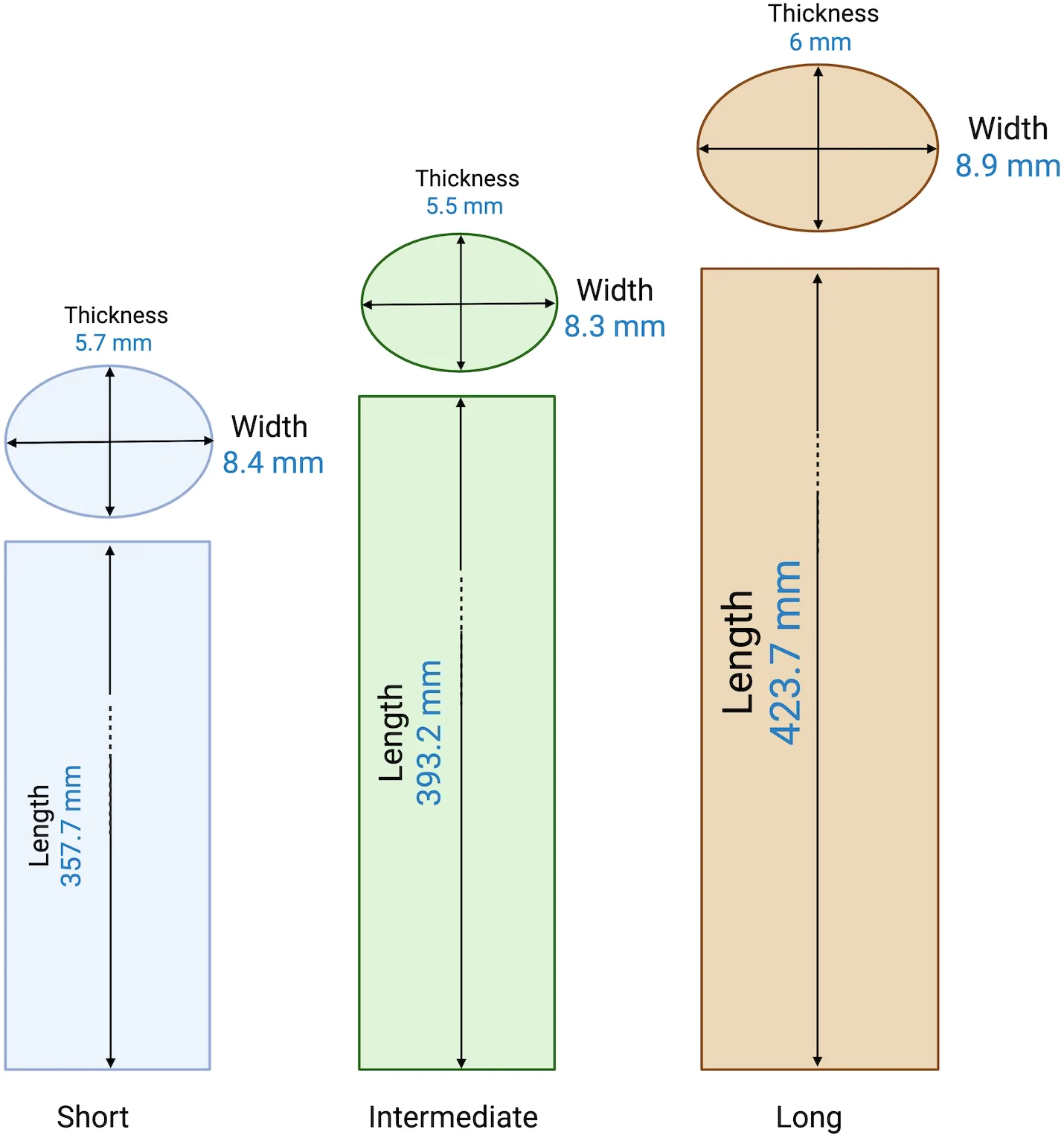
Schematic illustration of the 3 groups of spinal cords. The simplified representation shows the differential behavior of the average thickness and width of the 29 segments when switching from the Intermediate to the Long group or from the Intermediate to the Short group. While both transverse dimensions increase with an increase in total SC length in the first case, transverse dimensions do not decrease in the second case and even slightly increase. This suggests that the Short group may have a compensatory effect to overcome its smaller length, while maintaining function.

The correlation analyses by groups also highlighted the specificity of the L4 and C6 segments and brought more details. Although no clear tendency was found in the raw length data of the 3 groups, the segment coordinate method improved consistency between consecutive segments, demonstrated by strong correlations near the diagonal line (Figs 7−2, 3). In this case, scaling did not add much information, except a correlation increase in the thoracic region, maybe due to the correction and anatomical smoothing effect of the coordinate method. A small, correlated region between the first cervical segments and the L3-S3 segments in the *Short* group was also observed without clear explanation for the correlation of such distant segments.

Beyond these exploratory results, an important aspect to consider in the present study is the concept of segment, including its anatomo-clinical significance [15,16]. Although SC segments are anatomically purely descriptive entities rather than neurologically functional units, they remain the standard framework used in clinical practice. Histologically, there is no strictly definable unit based on transverse boundaries. Although spinal rootlets sometimes appear to define distinct levels, they actually cover overlapping territories between adjacent segments, reflecting the rostro-caudal distribution of the spinal nerves [15]. Despite these considerations, the segmental organization of the SC remains a practically valuable standard in clinical practice when precise localization of specific SC segments may be of importance for lesion diagnosis or functional prognosis. It is widely used in the American Spinal Injury Association (ASIA) impairment scale to localize an SC injury [17] or detect the progressive spread of neurodegenerative conditions such as amyotrophic lateral sclerosis (ALS) or tumoral processes. It may also provide a better understanding of the discrepancies between the clinical and vertebral levels in imaging or improve the targeting of electrical SC stimulation, currently based mainly on vertebral landmarks. Indeed, the common method of referring to a SC segment as being located behind the adjacent vertebral bodies is inaccurate and fails to account for interindividual variability [14]. In this context, an increasing number of MRI studies are attempting to precisely localize SC segments using rootlet tracking methods, without reference to vertebral elements [12,13]. Therefore, the results of this exploratory study may ultimately provide supplementary materials to guide clinicians in their daily routines.

The present study presented the limitations shared by every postmortem examination on which most human anatomy relies. First, the age and ethnic origin of the subjects were clearly influenced by the body donation process and can hardly be considered representative of the general population. Although few similar studies have a larger sample size, the relatively small number of subjects and the high variability in the *Short* and *Long* groups must also be considered a limiting factor, particularly in the *Short* group (n = 9). In addition, the division of the sample into three groups was based on a visual distinction rather than on an objective statistical classification. Therefore, future investigations should include larger sample size to challenge our observations and to achieve this aim, in vivo MRI studies are most appropriate.

Although subjects with neurological or neurodegenerative conditions were excluded, age-related alterations to the SC tissue may have existed, given that a previous study demonstrated a slightly smaller cross-sectional area in elderly subjects compared to younger ones [18]. Additionally, the embalming process with zinc chloride and fixation of the specimens in formaldehyde induced structural shrinkage, which may have affected the raw measurements. Direct measurements of three-dimensional fixation-induced deformation in the human SC are currently limited, but we assumed that fixation-related tissue shrinkage was approximately uniform. Although anisotropic deformation cannot be completely excluded we assume that patterns and correlations demonstrated in our results based on the scaling of the data that we proposed lead to a novel understanding of the human SC anatomy. Theoretically, this new approach would remain valid in *in vivo* measurement contexts, as the scaling strategy is inherently designed to limit size variations and thus shrinkage effects. Additionally, we acknowledge that the morphological modifications resulting from extracting the specimens, as well as the straightened state in which our measurements were obtained, do not reflect *in vivo* conditions, but is a necessary tool toward standardized data. Furthermore, one might consider this physical straightening comparable to the straightening method used in MRI to align the SC with the vertebral levels during the template generation [19], which smooths the very high variability observed in spine kyphosis, lordosis or rotation angles. However, further morphometric investigations should be undertaken to confirm these structural working hypotheses, and a correction factor should be determined between MRI and cadaveric measurements coming from the same subjects. Although practically challenging, this approach would ensure that our observations are valid and that our conclusion may reasonably be translated into a clinical MRI context.

Indeed, the concepts and results provided by our three consecutive morphometric studies [10,11] were designed to be ultimately adapted and applied to an MRI based dataset.

The present cadaveric findings provide an anatomical and methodological basis for future MRI-based validation studies and are an important step toward establishing normative references and creating a predictive model of the SC segment, currently under development.

## Conclusion

The present study defines a comprehensive system of analysis combining an original scaled length coordinate system, transverse dimension scaling, and sample grouping according to the total SC length. The transition from raw measurements to relative scaled data enabled us to reveal promising landmarks, corresponding to the L4 and C6 segments, likely the thickest area of the lumbar region and the widest area of the cervical region, respectively. By dividing the sample into 3 groups, we observed how average thickness and width seem to vary according to total SC length and we attempted to clarify how length fluctuations determine the behavior of these transverse dimensions. Altogether, these original observations unveiled unexpected and revealing features regarding our understanding of the human SC cadaveric anatomy, which should now be validated in an *in vivo* context. It also paves the way for building a probabilistic model that may precisely localize specific segments in the SC.

## Supporting information

S1 Fig Different localization methods to measure each SC segment. A: Schematic anterior view showing method used in previous studies [1]. The length of each spinal cord segment was defined as the root attachment length plus the upper inter-root length. B: Anterior view of SC segments marked with entomological needles according to the method illustrated in C. C: Method used in this study where a point was defined at mid-distance between the lowermost and uppermost rootlets of 2 consecutive spinal nerves. The intersection of a line drawn between these left and right points with the midline is considered the limit between two consecutive segments. Illustrations A and C adapted from https://sites.uclouvain.be/braininteratlas/en.
(TIF)
